# Mechanical and Thermal Evaluation of Aluminum Hybrid Nanocomposite Reinforced with Alumina and Graphene Oxide

**DOI:** 10.3390/nano11051225

**Published:** 2021-05-06

**Authors:** Abdul Samad Mohammed, Tawfeeq Saad Alahmari, Tahar Laoui, Abbas Saeed Hakeem, Faheemuddin Patel

**Affiliations:** 1 Department of Mechanical Engineering, King Fahd University of Petroleum and Minerals, Dhahran 31261, Saudi Arabia; tawfeeqalahmari@gmail.com (T.S.A.); faheemmp@kfupm.edu.sa (F.P.); 2 Interdisciplinary Research Center for Advanced Materials, KFUPM, Dhahran 31261, Saudi Arabia; 3 Department of Mechanical and Nuclear Engineering, University of Sharjah, Sharjah 27272, United Arab Emirates; tlaoui@sharjah.ac.ae; 4 Center of Research Excellence in Nanotechnology, King Fahd University of Petroleum and Minerals, Dhahran 31261, Saudi Arabia; ashakeem@kfupm.edu.sa

**Keywords:** aluminum, metal matrix, nanocomposites, graphene oxide, alumina, spark plasma sintering

## Abstract

Aluminum matrix composites are among the most widely used metal matrix composites in several industries, such as aircraft, electronics, automobile, and aerospace, due to their high specific strength, durability, structural rigidity and high corrosion resistance. However, owing to their low hardness and wear resistance, their usage is limited in demanding applications, especially in harsh environments. In the present work, aluminum hybrid nanocomposite reinforced with alumina (Al_2_O_3_) and graphene oxide (GO) possessing enhanced mechanical and thermal properties was developed using spark plasma sintering (SPS) technique. The focus of the study was to optimize the concentration of Al_2_O_3_ and GO content in the composite to improve the mechanical and thermal properties such as hardness, compressive strength, heat flow, and thermal expansion. The nanocomposites were characterized by FESEM, EDS, XRD and Raman spectroscopy to investigate their morphology and structural properties. In the first phase, different volume percent of alumina (10%, 20%, 30%) were used as reinforcement in the aluminum matrix to obtain (Al+X% Al_2_O_3_) composite with the best mechanical/thermal properties which was found to be 10 V% of Al_2_O_3_. In the second phase, a hybrid nanocomposite was developed by reinforcing the (Al + 10 V% Al_2_O_3_) with different weight percent (0.25%, 0.5%, 1%) of GO to obtain the optimum composition with improved mechanical/thermal properties. Results revealed that the Al\10 V% Al_2_O_3_\0.25 wt.% GO hybrid nanocomposite showed the highest improvement of about 13% in hardness and 34% in compressive strength as compared to the Al\10V% Al_2_O_3_ composite. Moreover, the hybrid nanocomposite Al\10 V% Al_2_O_3_\0.25 wt.% GO also displayed the lowest thermal expansion.

## 1. Introduction

Aluminum (Al) has traditionally been used for a variety of applications because of its lightweight, high corrosion resistance, high electrical, thermal conductivity and better formability compared to ferrous and other non-ferrous metals. However, the use of plain Al is generally not suitable for engineering applications because of its high ductility and low strength. This has led to the development of a variety of aluminum-based alloys with enhanced mechanical and tribological properties for a wide range of engineering applications. These alloys exhibit high strength to weight ratio, good machinability and lower cost of fabrication [1,2,3,4]. Further enhancement in the properties has been achieved by the development of aluminum metal matrix composites (MMCs) by adding different reinforcements [5]. The advantage of Al-MMCs is that they can be sintered with tailored properties by using a combination of various inclusions in the matrix [6]. Ceramic reinforcements such as SiC [7,8,9], TiO_2_ [10]_,_ Al_2_O_3_ [11,12,13,14,15] and a combination of various oxides, carbides and nitrides have been used to prepare Al-MMCs [16,17]. SiC reinforced Al composites have exhibited an increase in yield strength, tensile strength, hardness and density with the increase in the SiC content; however, with a decrease in toughness and plasticity [9,18]. SiC addition is reported to improve work hardening rate and work-to-fracture along with the ultimate strength of the composite [7]. Studies involving Al_2_O_3_ reinforcements have shown that density, hardness and wear resistance of Al–Al_2_O_3_ composite increase with increasing alumina content [15]; however, in other studies, the higher volume fraction of the Al_2_O_3_ reinforcement particles are reported to decrease the density of the composites. This discrepancy may be attributed to a difference in the shape and size of the reinforcement particles [13]. Recently, carbon-based reinforcements have been used to impart improvements in the properties of Al-MMCs [19]. Carbon materials such as carbon fibers [20,21], graphite [22,23], carbon nanotubes (CNTs) [24,25], graphene (G) [26,27] and graphene oxide (GO) [28,29] have been used as reinforcements in Al-MMCs. In graphene-reinforced aluminum matrix nanocomposites, an improvement of 79, 49 and 44% in yield strength, ultimate strength, and Vickers hardness have been reported with 1 wt.% GO addition, and the increase in GO content has led to grain refinement of the composite [30]. In another study, an increase in GO has yielded an increase in the hardness of Al-GO composites up to 0.4 wt.% GO, with a maximum increase of 163.8%, but the hardness has declined upon increasing the GO reinforcement to 0.6 wt.%. The wear rate of Al-GO composites is found to reduce with increasing GO content [31]. However, graphene is prone to forming aluminum carbide during the processing of Al-graphene composites, which lowers the hardness and tensile strength. This is attributed to the defective nature of graphene produced by thermal exfoliation/reduction of graphite. 

From the above literature review, it can be concluded that the use of monolithic reinforcement materials in Al-MMCs tends to have undesirable effects on some properties while enhancing other properties. For instance, Al-MMCs reinforced with ceramic inclusions have shown to improve the strength and stiffness, at the expense of ductility and fracture toughness [32,33]. To overcome these challenges, hybrid composites such as Al-MMCs containing both ceramic and carbon-based reinforcements are being developed [34,35,36,37]. Hybrid Al-SiC-GO prepared by stir casting method has shown a significant improvement in impact strength, tensile strength, hardness and wear resistance with the increase in the weight percentage of GO particles [38]. Similarly, Al-TiO_2_-GO hybrid composite with 10% TiO_2_ and various amounts of graphene (0.5, 0.75 and 1.0 wt.%) has exhibited an increase in hardness, ultimate tensile strength and wear resistance with increasing GO reinforcement. The selection of a processing method for Al-MMCs is shown to have an appreciable effect on the properties of hybrid Al-MMCs, which are generally sintered via powder metallurgy process, i.e., powder consolidation and sintering. The powder metallurgy process provides greater flexibility and controllability. Sintering is an important step that significantly affects the integrity and properties of composite materials sintered through powder metallurgy process. Conventional sintering is performed by heating the consolidated mixture of the powders in a furnace. However, nonconventional sintering techniques such as laser-assisted sintering, microwave-assisted sintering and spark plasma sintering are also being used. Spark plasma sintering (SPS) has shown several advantages over conventional sintering methods leading to marked improvements in the properties of materials. SPS integrates the consolidation and sintering stages and the material can be sintered with high heating rate and at relatively lower sintering temperature, thus leading to shorter processing time [39]. 

The review of literature has shown that studies on the development of hybrid Al-MMCs reinforced with ceramic-carbon materials are rather scarce, particularly Al-Al_2_O_3_-GO. Moreover, earlier studies have predominantly utilized the stir-casting method for consolidating the composites followed by conventional sintering. 

This has motivated the authors to sinter aluminum hybrid nanocomposite reinforced with alumina (Al_2_O_3_) and graphene oxide (GO) using spark plasma sintering (SPS) process, and to evaluate the effect of varying the amount of Al_2_O_3_ and GO on the mechanical and thermal properties of the developed hybrid nanocomposite.

## 2. Materials

Aluminum (Al) powder with a particle size of 30 µm and a purity of 99.5% was used as the matrix. It was procured from Alpha chemical company. Alpha alumina (Al_2_O_3_ ) powder manufactured by Union Carbide corporation for Buehler Ltd., Lake Bluff, IL, USA was used as a reinforcement with 300 nm particle size and 99.8% purity with a surface area of 85–115 m^2^/g. Graphene oxide used as a second reinforcement was procured from AD-Nano Company, Shimoga, India, with the following specifications: purity ~99%, surface area 250 m^2^/g. The XRF analysis of the as-received powders is shown in Table 1.

### SEM and XRD Analysis of the as Received Powders

The morphology of the as received powders was analyzed by scanning electron microscopy (SEM). X-ray diffraction (XRD) was also conducted to determine the phases of the as received powders. XRD was carried out on a Rigaku Miniflex X-ray diffractometer, using Cu Kα radiation (λ = 0.15416 nm) in the 2θ range 5°–120° at a scanning speed of 2 °/min. 

**Figure 1:**
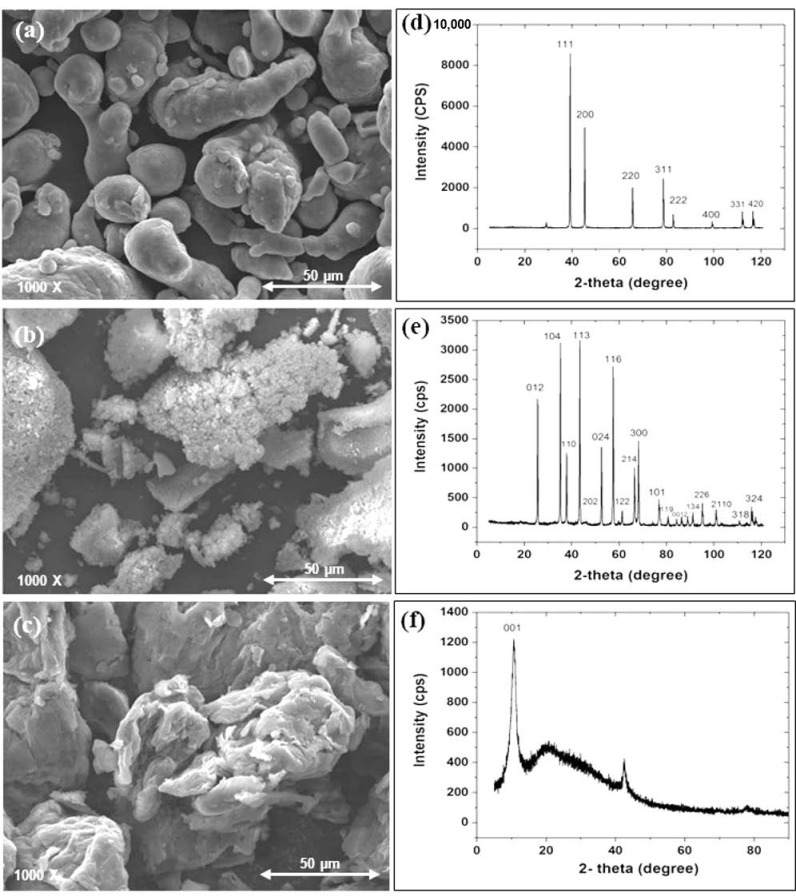
(**a**–**c**) SEM and (**d**–**f**) XRD for the as received powders of (**d**) Al, (**e**) Al_2_O_3_, (**f**) GO.

Figure 1a,c displays the high magnification SEM images of as received Al powder, Al_2_O_3_ and GO, respectively. It can be observed from Figure 1a that Al particles are spherically shaped with an average diameter of 30 μm. Figure 1b shows the Al_2_O_3_ particles that are acicular in shape and which gather to form agglomerates in some areas, whereas Figure 1c shows small sheets of GO. The XRD spectra of the as received powders are displayed in Figure 1d–f, indicating that they exhibit the signature peaks of the as received Al, Al_2_O_3_ and GO powders. 

## 3. Experimental Procedure

The steps for fabricating Al\X% Al_2_O_3_ nanocomposite and Al\X% Al_2_O_3_\Y% GO hybrid nanocomposite samples included ultrasonication, ball milling and spark plasma sintering. The different parameters used during each step are indicated in this section.

### 3.1. Ultrasonication of Al_2_O_3_ and GO Powders

Prior to mixing the reinforcements with the matrix Al powder, each of the reinforcements, namely, Al_2_O_3_ and GO were sonicated individually in ethanol for 10 min and 1 h, respectively using a probe sonicator (Sonics VCX 750, Newtown, CT, USA) at room temperature with an On\Off cycle of 20\5 s and an amplitude of 45%. Different volume percent (10%, 20%, 30%) of Al_2_O_3_ and different weight percent (0.25, 0.5 and 1 wt.%) of GO were sonicated under the same conditions to prepare different compositions.

### 3.2. Ball-Milling Procedure

Pure Al with different Al_2_O_3_ volume percent (10%, 20%, 30%) was loaded in zirconia vials and mixed for 24 h in a ball mill attritor (HD/HDDM/01, Union process, Inc. Akron, OH, USA) to produce a homogeneous mixture. The process was carried out under the flow of Argon (Ar) gas atmosphere to avoid oxidation. A total of 50 mL of ethanol was used as a process control agent (PCA) to avoid excessive cold welding and agglomeration. Zirconium oxide (ZrO_2_) balls with a diameter of 5 mm were used with a ball-to-powder weight ratio (BPR) of 10:1. Mixing was performed at a speed of 200 rpm. The ball milling experiment was halted after the first hour of the process to remove any powder from the walls of the vial to eliminate its accumulation on the walls. The vials were purged with Ar gas during the whole mixing process. Subsequently, the powder mixture was dried in an oven at a temperature of 80 °C for 12 h. The same procedure was used for 48 h to mix the Al\X% Al_2_O_3_\Y% GO hybrid powders to obtain a homogeneous mixture. Table 2 summarizes the mixing parameters used for the nanocomposites and the hybrid nanocomposite powders.

### 3.3. Spark Plasma Sintering Procedure

As-received Al powder was used to fabricate a reference sample. Al powder was charged in a 20 mm graphite die. A graphite sheet approximately 0.35 mm thick was placed in between the die and the powder as well as between the powder and the punch to easily remove the sample and avoid the wear of the punch. Spark plasma sintering machine from FCT group, System GMBH, (Rauenstein, Germany) was used to sinter the Al, the Al-X% Al_2_O_3_ nanocomposite and Al\X% Al_2_O_3_\Y% GO hybrid nanocomposite samples. In addition to the mentioned parameters in Table 3, the other SPS sintering parameters included, cooling rate = 100 °C/min to room temperature (20–35 °C), pulse = 1 ms, pause = 0, and number of pulse = 1. Circular samples of 20 mm diameter with a thickness of 6 mm were obtained after sintering. 

The sintered samples were mounted by using hot mounted and grounded using different grit papers starting from rougher to the finer grit (240, 320, 400, 600, 800, 1200) followed by polishing with 0.3 μm alumina paste to obtain a polished surface. The samples were subjected to ultrasonic cleaning for 10 min to remove any debris being subjected to further characterizations.

### 3.4. Densification, Mechanical, Thermal and Thermomechanical Analyses 

Various characterization techniques were used to evaluate the mechanical and thermal properties of the nanocomposites and the hybrid nanocomposites. Density measurements were carried out in line with the Archimedes principle (Kern ABT weighing scale, 320 g capacity, Balingen, Germany). Hardness measurements were carried out using a Zwick Roell Vickers hardness testing machine (Ulm, Germany) at a load of 500 gf. An average of 10 readings was taken for each sample. Scanning electron microscopy (SEM) fitted with an electronic dispersive x-ray (EDX) (Quanta FEG 250, Thermo Fisher company, Waltham, MA USA) was used to evaluate the morphology and the chemical composition of the samples. A compression test was carried out on an Instron testing machine to determine the behavior or response of the nanocomposites when exposed to compressive loads. Al is a soft material, and prone to dimensional instability due to its expansion when exposed to high temperature. A higher coefficient of thermal expansion indicates a more expansion tendency of the material. Hence, to evaluate the thermal expansion of the developed nanocomposites, Mettler Toledo instrument (TMA/SDTA LF/100, Columbus, OH, USA) was used for thermal expansion measurement.

## 4. Results and Discussion

The results are presented in three sub-sections. Firstly, the morphology and mechanical characterization results for the Al-X vol% Al_2_O_3_ composites are presented (Phase I) followed by the characterization of the hybrid nanocomposite (Phase II) and concluding with the mechanical and thermal characterization of the optimum hybrid nanocomposite (Phase III).

### 4.1. Results of Phase I-Morphology and Mechanical Characterization of Al-X vol% Al_2_O_3_ Nanocomposites

#### 4.1.1. SEM Analysis of Al-X%Al_2_O_3_ Nanocomposite Powders after Mixing

**Figure 2:**
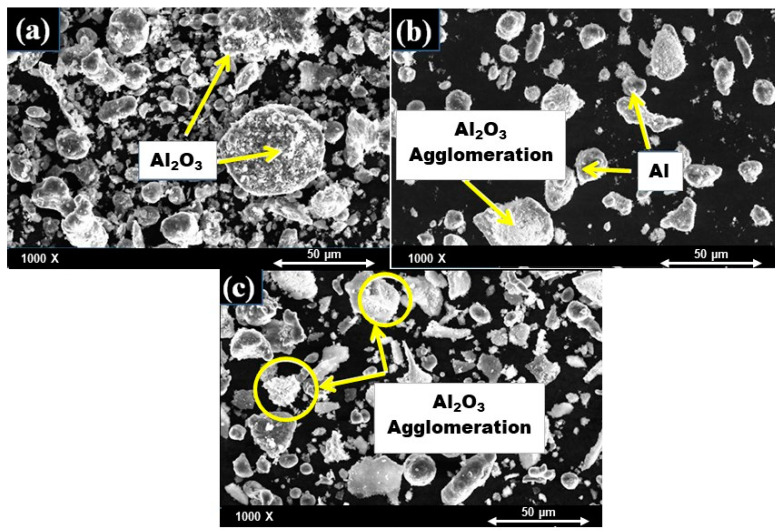
SEM images (backscattered) of Al mixed with (**a**) 10 vol%, (**b**) 20 vol%, and (**c**) 30 vol% Al_2_O_3_ powders.

The morphology of the nanocomposite powders of Al mixed with different volume percent of Al_2_O_3_ after ball milling was evaluated by SEM as shown in Figure 2a–c. It can be observed that in all nanocomposite powders, the particles deformed from a spherical shape into a relatively irregular shape after ball milling because of the collisions between the balls and the powder particles. It can be observed from Figure 2a that in Al-10% Al_2_O_3_, the nanoparticles of Al_2_O_3_ are uniformly distributed, whereas in Al-20%Al_2_O_3_ and Al-30% Al_2_O_3_ it can be observed the Al_2_O_3_ particles are non-uniformly distributed with a significant amount of agglomeration. The agglomeration tends to increase as the volume content of Al_2_O_3_ is increased from 20% to 30% Al_2_O_3_. Therefore, reducing the agglomeration would be a key element of improving the mechanical properties of Al- (20% and 30% Al_2_O_3_) nanocomposites due to the restriction of the interfacial area between the matrix and the reinforcement.

#### 4.1.2. Microstructure of (Al-X%Al_2_O_3_) Nanocomposite Samples after SPS

**Figure 3:**
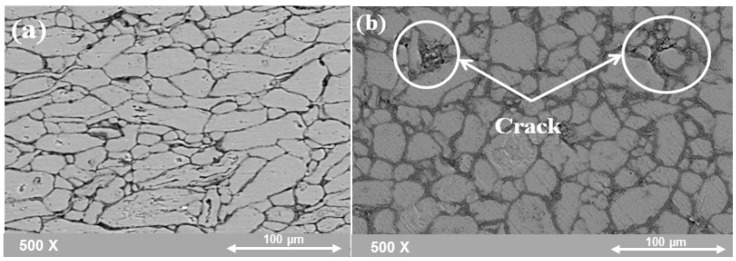
SEM images of the samples after SPS. (**a**) Al-10 vol% Al_2_O_3._ (**b**) Al-20 vol% Al_2_O_3._

Figure 3a, shows the SEM images for the SPS sample of Al-10 vol% Al_2_O_3_ nanocomposite, where little porosity can be observed with fine grain size. The samples were etched by buffered hydrofluoric acid (HF) for 10 s (1 mL HF and 49 mL water) for imaging. Adding more amount of Al_2_O_3_, as in the Al-20 vol% Al_2_O_3_ sample, Al_2_O_3_ is mainly observed along the grain boundaries of Al as illustrated in Figure 3b. A few cracks are also observed around the grain boundaries of the sample containing 20 vol% Al_2_O_3_. This can be attributed to a higher volume percent of Al_2_O_3_ content which makes the material brittle. Therefore, due to the brittleness of the sample which is associated with an increase in the volume percent of Al_2_O_3_, the fracture rate of the sample increased as was observed with Al-30 vol% Al_2_O_3_ which fractured during the grinding and polishing of the sample. Hence, the SEM could not be taken owing to the difficulty faced during grinding and polishing.

#### 4.1.3. Density of Al-X vol% Al_2_O_3_ Nanocomposites

**Figure 4:**
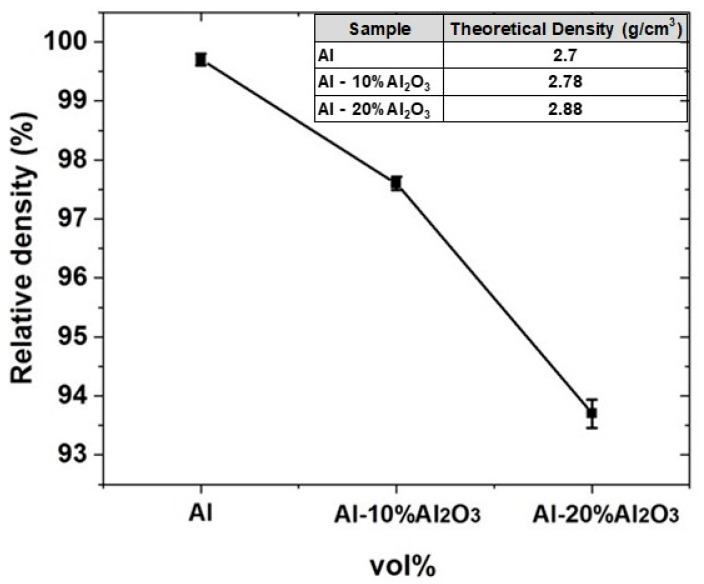
Relative density of Al, Al-10 vol% Al_2_O_3_, Al-20 vol% Al_2_O_3_. Inset: Theoretical densities of the different samples.

After sintering and grinding, Al-X vol%Al_2_O_3_ nanocomposite samples, the density was measured based on the Archimedes method and the results are shown in Figure 4. It is observed that Al displays a higher density of 99.7% as compared to the nanocomposite samples, with a density of 97.5% and 93.7% for Al-10 vol% Al_2_O_3_ and Al-20 vol% Al_2_O_3_, respectively. This density decrease can be attributed to the reduction in the wettability between Al_2_O_3_ and Al matrix due to the agglomeration of Al_2_O_3_ particles, particularly at high volume content. However, for the sample containing 30 vol% Al_2_O_3_, the density could not be measured because of the challenges mentioned above. The theoretical densities were measured using the rule of mixtures and are shown in Figure 4 (Inset).

#### 4.1.4. Hardness of Al-X vol% Al_2_O_3_ Nanocomposites 

**Figure 5:**
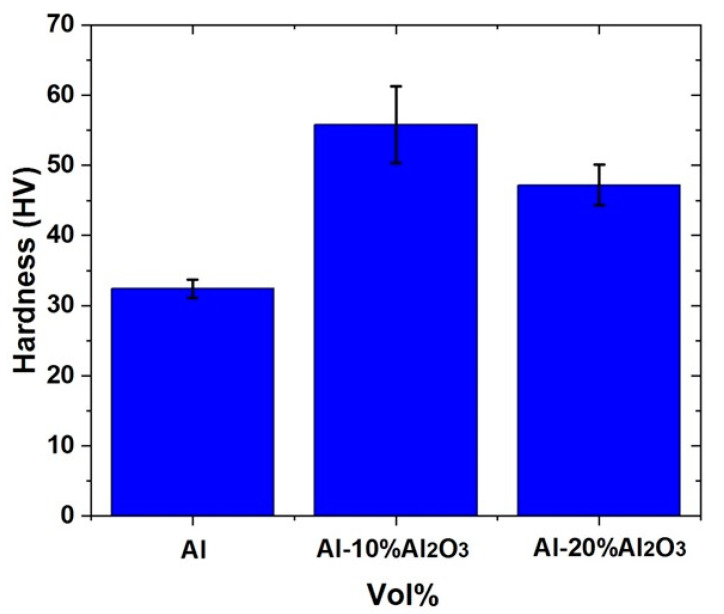
Variation of Hardness for Al, Al-10 vol% Al_2_O_3_, Al-20 vol% Al_2_O_3._

Figure 5 displays the hardness values of the sintered samples of Al-X vol% Al_2_O_3_ nanocomposite. The sintered Al sample showed a Vickers hardness value of 32 HV. However, the addition of 10 vol% of Al_2_O_3_ resulted in a significant increase in the hardness from 32 to 55.8 HV. This tremendous increase in the hardness can be attributed to the presence of the uniformly distributed hard and non-deformable nanoparticles of Al_2_O_3_ particles within the Al matrix, as can be seen in Figure 3a. The presence of these particles thereby hinders the movement of dislocations, resulting in an increase in the hardness. Further increasing the amount of Al_2_O_3_ to 20 vol% resulted in a reduction in the hardness from 55.8 to 47.2 HV. This reduction can be attributed to the lower densification triggered by the agglomeration of Al_2_O_3_ particles, as clearly observed in the SEM image Figure 3b owing to high volume percent and non-uniform distribution of Al_2_O_3_. As mentioned earlier, with a further increase in the volume percent of Al_2_O_3_ to 30%, the nanocomposite sample fractured due to an increased brittleness resulting from the agglomeration and cracks during sintering, due to which, the hardness measurements were not acquired. 

#### 4.1.5. Summary of Phase I

Based on the above results, Al-10 vol%Al_2_O_3_ nanocomposite showed the highest hardness, reasonable density and uniform distribution of Al_2_O_3_ particles in the Al matrix compared to the other developed nanocomposites. Hence, 10 vol% Al_2_O_3_ was selected as a first filler to fabricate the Al hybrid nanocomposite. 

### 4.2. Results of Phase II-Morphology and Mechanical Characterization of Al-10 vol% Al_2_O_3_ –Y wt.% GO Nanocomposites

In this phase, Al hybrid nanocomposites were fabricated by reinforcing the Al-10 vol% Al_2_O_3_ with different weight percentages (0.25, 0.5 and 1 wt.%) of GO.

#### 4.2.1. SEM Analysis of Al-10 vol%Al_2_O_3_-Y wt.%GO Hybrid Nanocomposite Powders after Ball Milling 

**Figure 6:**
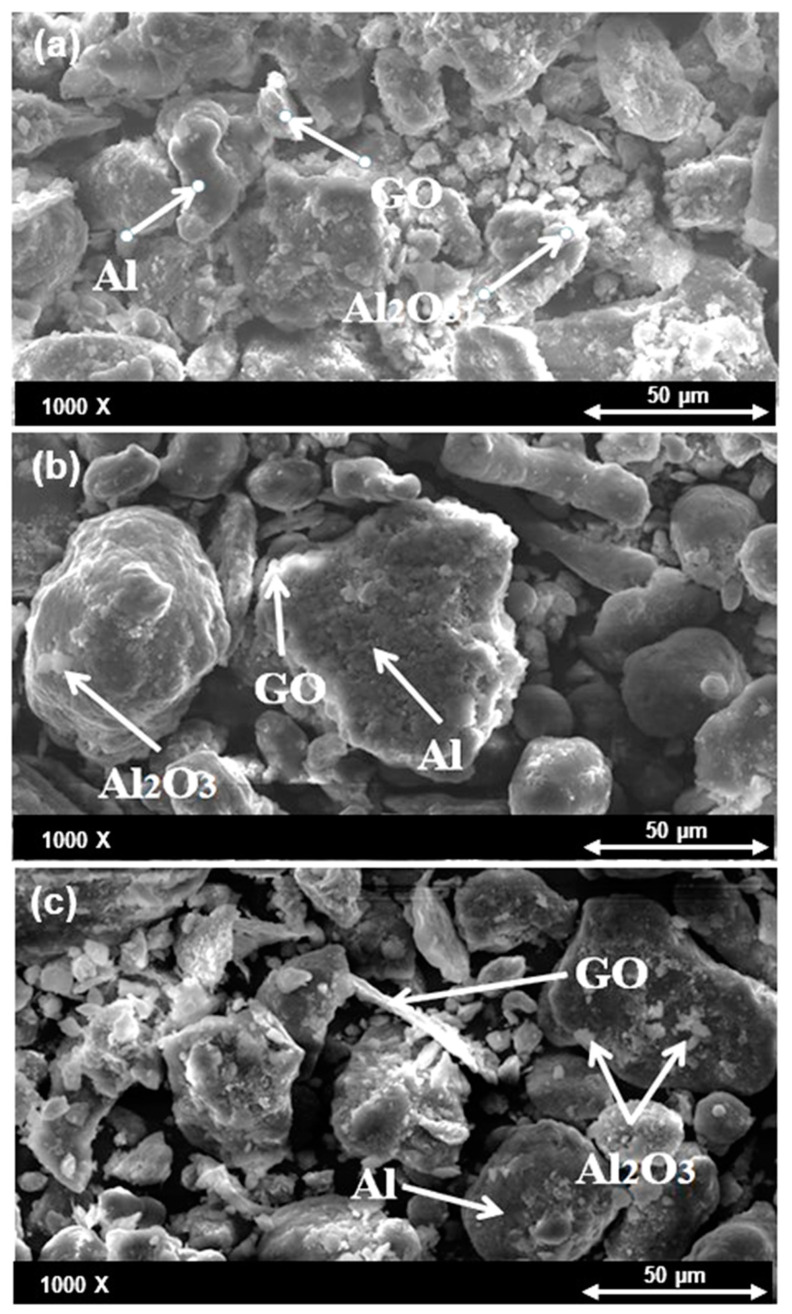
SEM images (backscattered) of the powders after mixing for (**a**) Al-10% Al_2_O_3_-0.25% GO. (**b**) Al-10% Al_2_O_3_-0.5% GO. (**c**) Al-10% Al_2_O_3_-1%GO.

Figure 6a–c represents the morphology of the mixed powders for the developed Al-10 vol% Al_2_O_3_- Y wt.%GO hybrid nanocomposite samples. A uniform distribution of GO can be observed for the Al-10 vol% Al_2_O_3_- 0.25 wt.%GO hybrid powders. 

#### 4.2.2. Microstructure of Al-10 vol%Al_2_O_3_-Y wt.%GO Hybrid Nanocomposites after SPS

**Figure 7:**
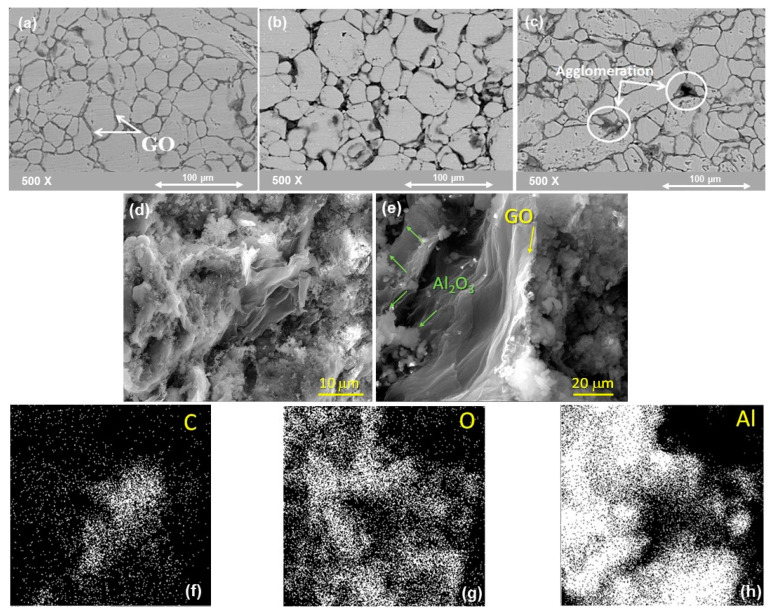
**A** SEM of SPS samples (**a**) Al-10% Al_2_O_3_-0.25% GO (**b**) 10% Al_2_O_3_-0.5% GO (**c**) 10% Al_2_O_3_-1 % GO. (**d**,**e**) from the fractured sample (Al-10% Al_2_O_3_-1%GO). (**f**–**h**) EDS-mapping for the fractured sample (Al-10% Al_2_O_3_-1%GO) revealing the distribution of Al_2_O_3_ of the grain boundaries of Al hybrid.

The microstructure of the developed hybrid nanocomposite was investigated by SEM as presented in Figure 7. A uniform distribution of GO along the grain boundaries is observed in the sample containing 0.25 wt.% GO, whereas, in the samples containing 0.5wt.% and 1 wt.% of GO some agglomeration of GO was observed as highlighted in Figure 7a. Another observation noted from Figures is the presence of porosity which is associated with the sample containing 1 wt.% of GO. 

Figure 7d,e SEM from the fractured sample and mapping of the sample confirms the relatively uniform distribution of the alumina particles in the aluminium matrix with 1 wt.% of GO hybrid. It was comprehended that finer alumina particles were trapped between the large aluminium particles, which resulted in better densification in the absence of GO. SEM image of hybrid shows the homogeneous dispersion of GO, as per fractured surface. The GO was embedded between the aluminium particles, as shown in the fractured surfaces, which promote the improvement in the toughness of the hybrid. SEM/EDS mapping micrographs, Figure 7f–h revealed the presence of both intergranular and transgranular fracture morphologies in the hybrid sample. Moreover, there are few regions where agglomerates were found which deteriorate and have an adverse effect on the properties of the hybrid sample.

#### 4.2.3. Density Measurement of Al-10% Al_2_O_3_-Y% GO Hybrid Nanocomposites

**Figure 8:**
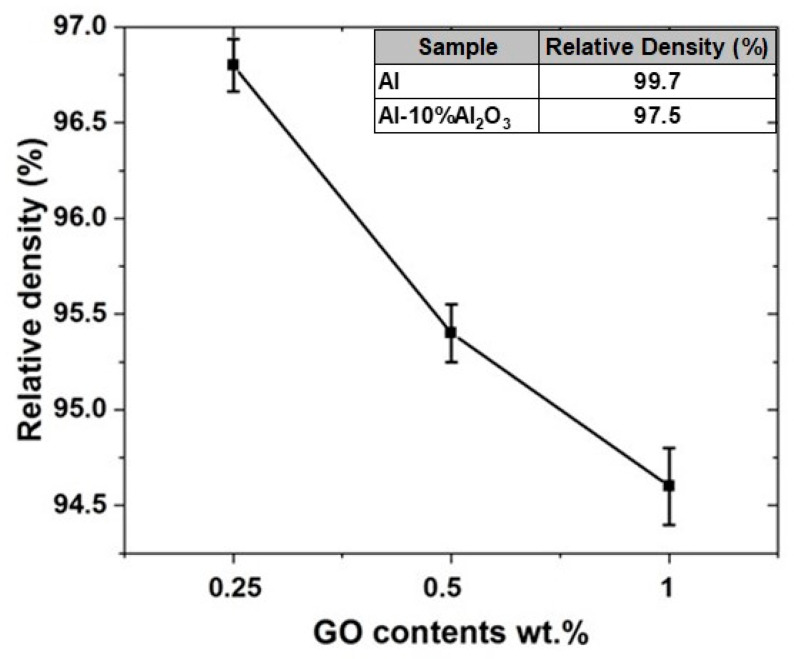
Relative density for Al-10% Al_2_O_3_-Y% GO hybrid nanocomposite Inset: Relative densities of pure Al and Al-10% Al_2_O_3._

After sintering and grinding/polishing of Al-10% Al_2_O_3_-Y wt.% GO hybrid nanocomposite samples, the experimental density was measured based on the Archimedes method and the results are shown in Figure 8. The results indicate that the relative density is reduced with increasing GO content, whereby, adding 0.25 wt.% of GO to the Al-10% Al_2_O_3_ nanocomposite decreases the relative density from 97.5% to 96.8%. Further addition of GO to Al-10% Al_2_O_3_ nanocomposite gradually reduces the relative density to 95.4% and 94.6% corresponding to 0.5 wt.% and 1 wt.% GO content, respectively. This reduction in the density is attributed to the tendency of GO to distribute itself along the grain boundaries which impedes the densification process, consequently resulting in higher porosity with a higher content of GO as observed in SEM images in Figure 7.

#### 4.2.4. Hardness Measurement of Al-10% Al_2_O_3_-Y% GO Hybrid Nanocomposites

**Figure 9:**
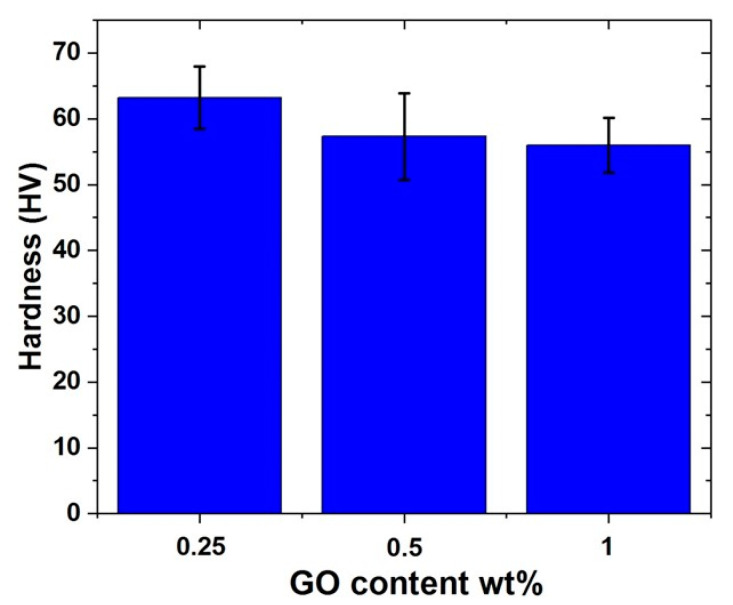
Hardness results for (Al-10% Al_2_O_3_- Y% GO) hybrid nanocomposites.

The hardness results of Al-10% Al_2_O_3_-Y% GO are presented in Figure 9. The highest hardness of 63 HV was observed for the hybrid sample containing 0.25 wt.% of GO among all the developed samples. The hardness reduced to 57 HV with an increase in the GO content to 0.5 wt.%. However, not much difference was observed in the hardness of the hybrid sample with a further increase in the GO content to 1 wt.%. The increase in the hardness of the hybrid nanocomposite with a low content of GO (0.25 wt.%) is attributed to the uniform distribution of both fillers, Al_2_O_3_ and GO, in the matrix. The homogeneous distribution of these fillers helps in the load transfer from the matrix leading to a higher hardness of the hybrid nanocomposite. Furthermore, these fillers as discussed above and shown by SEM, influence the microstructure of the hybrid nanocomposites resulting in finer grain size. The reason for observing relatively lower hardness in the samples containing 0.5 wt.% and 1 wt.% as compared to 0.25 wt.% GO sample is attributed to the low densification associated with the Al_2_O_3_ agglomeration and porosity.

#### 4.2.5. Raman Spectroscopy of Al-10% Al_2_O_3_-Y% GO Hybrid Nanocomposite Powders

**Figure 10:**
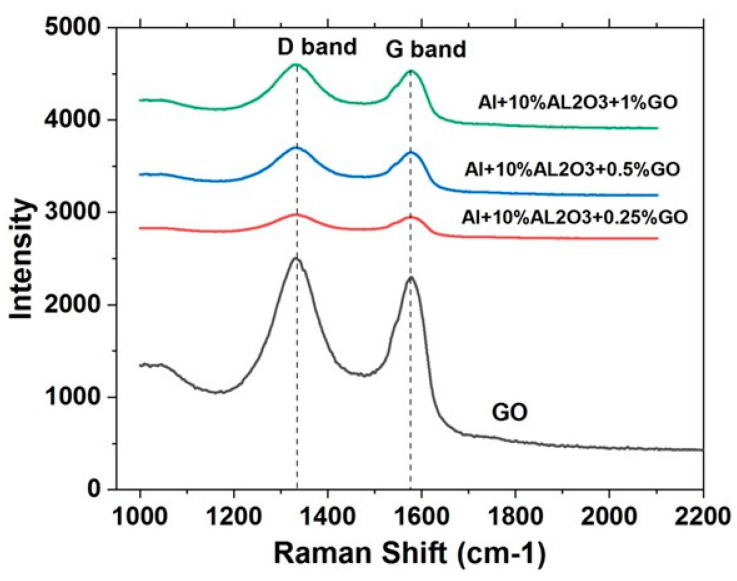
Raman spectroscopy for GO, Al-10% Al_2_O_3_-0.25%GO, Al-10% Al_2_O_3_-0.5%GO, and Al-10% Al_2_O_3_-1%GO hybrid nanocomposites samples.

Figure 10 shows Raman spectra of GO powder and the hybrid nanocomposite powders after mixing. It can be observed that GO shows two signature peaks/bands. One at approximately 1580 cm^−1^ corresponding to the G band resulting from the stretching of the C–C bond in GO, and another one at approximately 1350 cm^−1^, corresponding to the D band which is associated with the disorders or defects that occur from the resonance Raman spectra of Sp2 hybridized carbon. Both peaks can be observed in the Raman spectra for all the hybrid nanocomposite powders. However, their intensity increases with an increase in the GO content. 

#### 4.2.6. XRD Analysis of the Nanocomposite and Hybrid Nanocomposite Samples 

**Figure 11:**
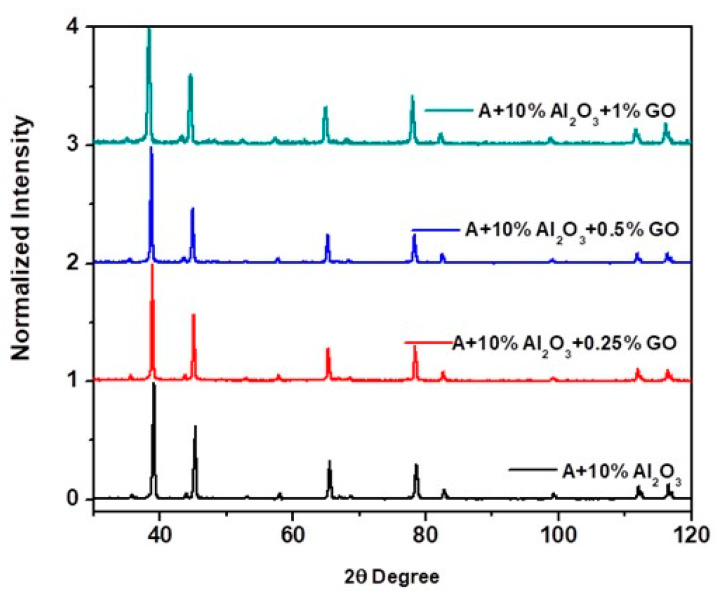
Normalized XRD spectrums the developed nanocomposite samples (Al-10%Al_2_O_3_) and hybrid nanocomposite (Al-10%Al_2_O_3_-0.25, 0.5, 1%GO).

Figure 11 shows the X-ray diffraction pattern obtained for the SPS sintered samples for Al-10%Al_2_O_3_ nanocomposites and all the developed hybrid nanocomposite samples. The XRD pattern of Al-10% Al_2_O_3_ nanocomposite shows slightly less broadened peaks for both Al_2_O_3_ and Al, as compared to the hybrid nanocomposite samples. This can be attributed to the effect of ball milling time where the nanocomposite was milled for 24 h while the hybrid nanocomposite powders were milled for 48 h resulting in a more homogeneous and uniform distribution of the fillers in the Al matrix. However, GO phase was not observed in the hybrid nanocomposite due to its very small amount. Moreover, it was observed that no chemical reaction occurred between GO and Al- Al_2_O_3_ nanocomposite as there was no new phase such as intermetallic phase(s) nor the formation of aluminum carbide (Al_4_C_3_) was seen in any of the XRD patterns for the hybrid nanocomposites.

The XRD patterns were normalised and the crystallite size and microstrain were also estimated by using the following equations: crystallite size (average in Å) = Kλ/(Bcosθ) and BT = CεTanθ (*ca*. C = 4 for spherical particles), respectively. The average crystallite size was found to be slightly decreasing, with an increased ball milling duration and the average microstrain is found to slightly increase for all compositions. The slight increase in microstrain value can be attributed to the presence of hard alumina particles and the cold welding of soft aluminum. 

#### 4.2.7. Summary of Phase II

From the above results, it can be concluded that the hybrid nanocomposite sample containing 0.25%GO showed the highest hardness, density and uniform distribution of the fillers in the Al matrix. Hence, based upon the above results Al-10% Al_2_O_3_-0.25%GO was selected for further processing.

### 4.3. Results of Phase III-Mechanical and Thermal Characterization of Al-10 vol% Al_2_O_3_–0.25 wt.% GO Hybrid Nanocomposites

Mechanical and thermal characterization of the optimum hybrid nanocomposite which was obtained from Phase II to be Al-10 vol% Al_2_O_3_-0.25 wt.% GO were evaluated. Compressive strength, differential scanning calorimetry and thermal expansions for the hybrid nanocomposite were investigated and presented below.

#### 4.3.1. Evaluation of Compressive Strength for Al, (Al-10% Al_2_O_3_) and (Al-10% Al_2_O_3_-0.25%GO) Nanocomposites

**Figure 12:**
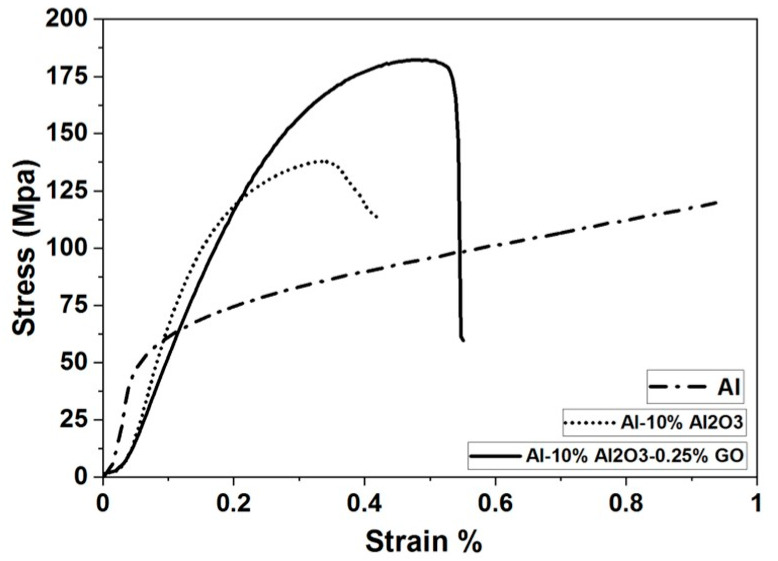
Compression test results for Al, Al-10% Al_2_O_3_ and Al-10% Al_2_O_3_-0.25% GO.

The results of compressive strength for Al as a reference matrix, Al-10% Al_2_O_3_ and the Al-10% Al_2_O_3_- 0.25%GO hybrid nanocomposite are presented in Figure 12. The compressive strength of Al sample measured to be 75MPa significantly increased to 130 MPa for the Al-10% Al_2_O_3_ nanocomposite, whereas the compressive strain reduced to 0.4% as compared to the Al sample. This effect could be attributed to the presence of the reinforcing Al_2_O_3_ hard nanoparticles. Moreover, the compressive strength further increased in the Al-10% Al_2_O_3_-0.25%GO hybrid nanocomposite to values of 180 MPa, about 30% higher than that of Al-10% Al_2_O_3_ nanocomposite. This improvement can be attributed to the presence of the uniformly distributed GO filler in the hybrid nanocomposite, leading to an improvement in the interfacial adhesion between Al_2_O_3_ and Al without overlapping or agglomeration. However, an increase in the compressive strain for the hybrid composite sample was observed to a value of about 0.52% as compared to the 0.4% compressive strain for Al-10% Al_2_O_3_ nanocomposite. This can be attributed to the structure of GO, which contains hydroxide (OH ^-^) and (O ^-^) chains, which in turn, leads to an increase in the length of the C–C bond at each hexagonal lattice as reported by Pop et al. [40]. These bonds will be dominant over Vander Waals attractions in (Al-10%Al_2_O_3_-0.25%GO) hybrid nanocomposites, thus results in strain improvement caused by the efficient load transfer from soft Al matrix to hard GO [41].

#### 4.3.2. Thermal Expansion Measurement for Al, (Al-10% Al_2_O_3_) and (Al-10% Al_2_O_3_-0.25%GO) Nanocomposites

**Figure 13:**
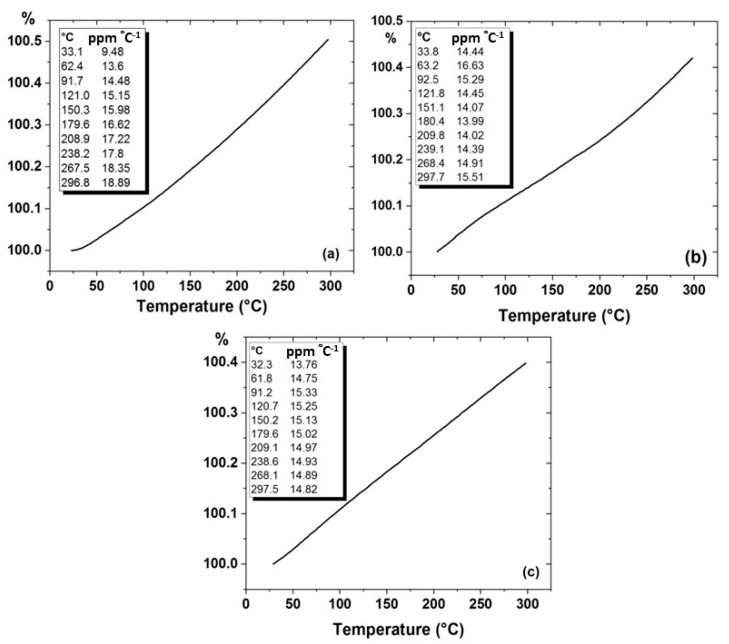
Thermal expansion of (**a**) Al, (**b**) Al-10% Al_2_O_3_, (**c**) Al-10% Al_2_O_3_-0.25% GO.

Thermal expansion was carried out for the developed SPS samples, and the results obtained are presented in Figure 13. It is observed that the coefficient of thermal expansion linearly increased with increasing temperature for all the developed samples. The thermal expansion of Al which is the reference matrix was found to be 18.89 ppm °C^−1^, whereas for Al-10% Al_2_O_3_ nancomposite it reduced to 15.51 ppm °C^−l^, leading to a reduction of 17%. Likewise, the thermal expansion further decreased for the Al-10% Al_2_O_3_-0.25%GO hybrid nanocomposite to a value of 14.82 ppm °C^−1^, leading to a reduction of 4.4% in the coefficient of thermal expansion as compared to Al-10% Al_2_O_3_ nanocomposite and reduction of 21% as compared to Al. 

Generally, the thermal expansion decreased as alumina and GO was incorporated into the matrix. Thermal expansion of metal matrix composites is strongly influenced by voids and the breaking of bonds between constituents of the composite. Increasing the alumina and GO content of the composite coincided with the appearance of voids and porosity resulting in reduced effective thermal strain and lowering the coefficient of thermal expansion of the composite [42]. The thermal expansion of a composite is dominated by the component that has the most outstanding bulk modulus value. The bulk modulus values of aluminium and sintered alumina have been reported to be 62 GPa and 257 GPa, respectively. Additionally, indeed, we observed the lowest thermal expansion values for compositions having the highest tested alumina and GO content and this relationship was likely due to alumina and GO inclusions having a relatively much lower thermal expansion value and high bulk modulus as compared to the aluminium matrix. 

#### 4.3.3. Summary of Phase III

From the above results, it can be concluded that the compressive strength was the highest in the hybrid nanocomposite (Al-10% Al_2_O_3_-0.25%GO) with a value of 180 MPa compared to (Al-Al_2_O_3_) (130 MPa) and Al (75 MPa). Heat flow and area associated with hybrid nanocomposite presented the lowest values as compared to Al and Al-10%Al_2_O_3_ nanocomposite. On the other hand, the coefficient of thermal expansion was the lowest for the hybrid nanocomposite sample. Table 4 presents a summary and comparison of thermal and mechanical properties obtained in the present research for the hybrid sample to the mechanical and thermal properties of other developed aluminum composites in the literature.

## 5. Conclusions

Hybrid aluminum nanocomposites reinforced with alumina and graphene oxide were successfully produced by powder metallurgy technique and spark plasma sintering. The study was conducted in three phases whereby, in phase1, the optimum volume percent of alumina content out of 10%, 20%, 30% was determined. It was found that Al reinforced with 10 vol% Al_2_O_3_ resulted in the best mechanical properties due to the uniform dispersion of Al_2_O_3_ particles throughout the Al matrix as observed in SEM micrographs. In phase 2, different weight percent of GO (0.25%, 0.5% and 1%) were added to Al-10% Al_2_O_3_ to form a hybrid nanocomposite. It was found that Al-10vol% Al_2_O_3_-0.25 wt.% GO resulted in the best mechanical properties in terms of hardness. The distribution of GO was identified to be along the Al grain boundaries while the Al_2_O_3_ particle were distributed between the grain boundaries of Al grains. XRD results confirmed that no chemical reaction or intermetallic phase was formed in Al-10% Al_2_O_3_ and Al-10% Al_2_O_3_-0.25%GO. The optimized hybrid nanocomposite was further characterized in phase 3 by measuring its compressive strength and its thermal expansion. It was found that adding 0.25 wt.% of GO into Al-10vol% Al_2_O_3_ nanocomposite increased the compressive strength by 30%. Moreover, the Al-10%Al_2_O_3_-0.25%GO hybrid nanocomposite showed the lowest coefficient of thermal expansion.

## Figures and Tables

**Table 1:** XRF analysis of the as received powders.

Material	Content	wt.%
**Aluminum (Al) Powder**	Al	>99.5
	Si	<0.25
	Fe	<0.15
	Ti	<0.25
**Alumina Powder**	Al_2_O_3_	99.88
	SiO_2_	0.034
	P_2_O_5_	0.0085
	S	0.026
	K_2_O	0.027
	TiO_2_	0.0022
	V_2_O_5_	0.0041
	MnO	0.0016
**Graphene Oxide (GO)**	C	77
	O_2_	22
	Other	1

**Table 2:** Ball milling parameters used for mixing the nanocomposite and the hybrid nanocomposite powders.

Material	Speed (RPM)	BPR	Mixing Time (Hours)	PCA	Atmosphere
**Al\X% Al_2_O_3_ nanocomposite powder**	200	10:1	24 h	Ethanol	Argon
**Al\X% Al_2_O_3_\Y% GO hybrid nanocomposite powder**	200	10:1	48 h	Ethanol	Argon

**Table 3:** Spark plasma sintering parameters used for preparing the nanocomposite and the hybrid nanocomposite samples.

Material	Temp (°C)	Pressure (MPa)	Holding Time (min)	Heating Rate (°C/min)
**Al\X% Al_2_O_3_ nanocomposite powder**	550	50	10	200
**Al\X% Al_2_O_3_\Y% GO hybrid nanocomposite powder**	550	50	10	200

**Table 4:** Summary of physical/mechanical and thermal properties of aluminum composites in the literature.

Material	Synthesis Method	Density (%)	Hardness (HV)	Tensile Strength (TS)/Compressive Strength (CS MPa)	Thermal Expansion (ppm °C^−1^)	Reference
Al–0.2 wt.% GO	Uniaxial compaction + tube furnace sintering	−	36	−	−	[29]
Al–4 wt.% Cu - 1 wt.% GO	SPS	99.26	125	320 (TS)	−	[30]
Al–7 wt.% Al_2_O_3_	SPS	93.6	38.77	-	−	[43]
Al–10% Al_2_O_3_-0.2% Graphene	In situ melt casting	−	198	79.91 (TS)	−	[44]
Al–10 wt.% Al_2_O_3_	SPS	95.58	85	65 (TS)	−	[45]
Al-7 wt.% BN	SPS	−	134	170 (TS)	−	[46]
Al-10 wt.% Al_2_O_3_	Uniaxial compaction + tube furnace sintering	98.1	81	-	−	[15]
Al-20 wt.% TiB_2_	SPS	96	180	540 (TS)	−	[47]
Al-10%Al_2_O_3_-0.25%GO	SPS	96.8	63.2	184(CS)	14.82	Present research

## Data Availability

The data presented in this study are available on request from the corresponding author.
